# Hierarchical detection of neonatal brain pathologies using a two-stage deep learning framework from magnetic resonance imaging

**DOI:** 10.3389/fnimg.2026.1981436

**Published:** 2026-10-30

**Authors:** Christian Ahwoi Ampiah, Tina Malalanirainy, Alessandro Crimi

**Affiliations:** 1 African Institute for Mathematical Sciences, Accra, Ghana; 2 Department of Computer Science, AGH University of Kraków, Kraków, Poland

**Keywords:** 3-D ResNet, brain-age gap, comorbidity detection, deep learning, hierarchical classification, hydrocephalus, hypoxic-ischaemic encephalopathy, myelination delay

## Abstract

Automated neonatal brain pathology detection from magnetic resonance imaging (MRI) faces a dual challenge: preterm birth produces a characteristic but confounding pattern of delayed myelination that can obscure concurrent structural abnormalities, while conditions such as hypoxic-ischaemic encephalopathy (HIE) and hydrocephalus demand timely identification in their own right. Existing frameworks typically address these clinical questions in isolation or collapse them into a single flat classifier, discarding the natural clinical decision hierarchy. This study presents a hierarchical two-stage deep learning pipeline that emulates clinical reasoning from paired T1-weighted and T2-weighted neonatal brain MRI. An initial Stage-1 deploys a brain-age gap classifier, built on a 3-D ResNet-18 regressor followed by a gap-thresholded binary decision intended to screen for myelination delay. Then the same subjects advance to a Stage-2, where a second 3-D ResNet-18, trained for structural pathology, performs three-class classification: *normal, HIE*, and *hydrocephalus*. Experiments demonstrate that a regression from MRI scan to age with an *R*^2^ of 0.941 at Stage-1, the proposed hierarchical pipeline achieves a Stage-2 area under the curve (AUC) of 0.919 ± 0.024.

## 1 Introduction

The neonatal brain undergoes rapid and coordinated developmental changes during the perinatal period (Nielsen et al., 2023). Among the most radiologically informative features there is myelination, which can be defined as progressive insulation of axonal fibers by oligodendrocyte-derived myelin, which produces well-characterized signal inversions on T1- and T2-weighted MRI that experienced neuroradiologists use to gauge neurological maturity (Chiu et al., 2026). Disruptions to this maturational timeline arise from a heterogeneous set of causes, broadly divisible into two clinical phenotypes that frequently co-occur yet demand distinct therapeutic responses.

The first phenotype is myelination delay associated with preterm birth. Infants delivered before 37 weeks of gestation exit the uterine environment before the rapid myelination surge of the third trimester (Back, 2015). On MRI, their brains appear structurally immature relative to their chronological (post-natal) age, a discrepancy readily captured by the brain-age gap δ = â−*a*, where â is a deep-network's myelin-informed age prediction and *a* is chronological age. A negative δ has been reported as a signature of preterm myelination delay (Liu et al., 2024).

The second phenotype encompasses structural pathologies, notably hypoxic-ischemic encephalopathy (HIE) and hydrocephalus, which produce abnormalities in cortical architecture, white matter signal, or ventricular morphology independently of gestational maturity (Counsell et al., 2008). HIE, caused by perinatal asphyxia, is the leading cause of neonatal brain injury in term infants and requires rapid identification because therapeutic hypothermia is effective only within a narrow treatment window (Shankaran et al., 2005). Hydrocephalus, characterized by pathological CSF accumulation and ventricular dilation, is often diagnosed incidentally on MRI and requires neurosurgical planning (Rekate, 2011).

A clinically important complication is that these two phenotypes can *co-occur*. Preterm infants are at elevated risk of both HIE and post-hemorrhagic hydrocephalus (Soul et al., 2007), meaning that a flat classifier must simultaneously navigate myelination-related and structure-related image features, an inherently ambiguous task. When myelination delay is absent, the differential diagnosis contracts to structural abnormalities alone, providing a cleaner signal. Mirroring this reasoning, this study proposes a hierarchical two-stage pipeline that first screens for myelination delay and then, conditional on its absence, performs fine-grained structural classification.

The specific contributions of this study are as follows:

1. Neonatal brain pathology detection is formulated as a hierarchical binary-then-ternary decision problem, decomposing a complex four-class task into two clinically motivated sub-problems that are each easier to solve than the joint problem.
2. A gap-based Stage-1 binary classifier is trained that routes subjects to a *preterm* label when the brain-age gap computed from a dual-modality 3-D ResNet-18 falls below a data-driven threshold, and to structural analysis otherwise.
3. A Stage-2 structural comorbidity classifier is trained, a second 3-D ResNet-18 is trained from random initialization that discriminates normal, HIE, and hydrocephalus, with Stage-1 flag ensuring preterm subjects are excluded from the final structural prediction.

The remainder of this paper is organized as follows. Section 2 reviews related work on brain-age modeling, neonatal pathology classification, and hierarchical neural networks. Section 3 describes the dataset, preprocessing, model architectures, and training protocol. Section 4 presents quantitative results and ablations. Section 5 discusses clinical implications and limitations. Section 6 concludes.

## 2 Related work

### 2.1 Brain-age estimation and the brain-age gap

Franke et al. (2010) is one of the first studies focused on brain-predicted age difference using relevance vector regression on structural MRI morphometrics. Cole et al. (2018) demonstrated that a larger brain-age gap is associated with increased all-cause mortality in adult cohorts, establishing the gap as a robust biomarker beyond simple age regression. In the neonatal context, Chiu et al. achieved sub-week accuracy in brain-age estimation from diffusion MRI on the dHCP cohort. More recently, Chen et al. (2022) trained a 3-D convolutional network to predict neonatal and infant brain age directly from myelination patterns on paired T1w/T2w MRI, closely paralleling Stage-1 design adopted here, while Fang et al. (2024) compared structural and diffusion-MRI-based brain-age prediction in neonates. A 2025 systematic review Asma Ull et al. (2026) surveys the rapidly expanding set of deep-learning approaches and open datasets for developmental brain-age estimation, underscoring the continued relevance of the brain-age-gap paradigm to neonatal neuroimaging. This study follows this lineage but repositions the brain-age gap as a *gating signal* for downstream structural classification rather than an end-product biomarker.

### 2.2 Preterm brain MRI analysis

Preterm birth is the most common context for neonatal neuroimaging. Bouyssi–Kobar et al. (2016) compared third-trimester volumetric brain growth in very preterm infants and healthy in utero fetuses, demonstrating gestational-age-dependent differences in global and regional brain growth. Wagner et al. (2022) subsequently applied MRI-based radiomics to T1- and T2-weighted images from very preterm neonates to predict later neurodevelopmental outcomes. A recurring challenge in all these studies is the confounding of prematurity-related myelination delay with structural injury. The present hierarchical design addresses this by treating myelination delay as a separate first-pass decision (Scheinost et al., 2023).

### 2.3 HIE and hydrocephalus detection

Automated HIE detection from MRI has been approached with random-forest lesion detection on diffusion-weighted imaging (Murphy et al., 2017) and, more recently, deep learning (Tian et al., 2023; Rajeshwari et al., 2025). More recent studies have continued to advance both conditions individually. Tian et al. (2023) combined structural MRI with clinical variables in a deep-learning clinical-radiomics nomogram, reporting a marked AUC drop from training (0.87) to independent external validation (0.80); Lew et al. (2024) trained a multisequence (T1w/T2w/DTI) deep-learning model on the 17-institution HEAL trial cohort to predict the 2-year neurodevelopmental outcome, explicitly evaluating out-of-distribution generalization across held-out institutions. A 2025 systematic review (Rajeshwari et al., 2025) of AI-driven neonatal MRI interpretation for HIE reported sensitivity ranging from 83 to 95% and specificity from 86% to 93% across five included studies. For hydrocephalus, Xu et al. (2025) introduced an open, expert-annotated pediatric hydrocephalus MRI dataset with choroid-plexus labeling, and Mukherjee et al. (2026) proposed a global-local parts-aware learner to differentiate healthy, post-infectious, and non-post-infectious hydrocephalus from low-field infant MRI. To the best of knowledge, no prior study has jointly framed HIE and hydrocephalus as structural comorbidities to be classified together for myelination delay.

### 2.4 Hierarchical and multi-stage classification

Hierarchical classification, implemented as a tree of binary or low-order decisions, has a long history in computer vision (Yan et al., 2015) and medical imaging (Shen et al., 2017), though it should not be confused with hierarchical clustering (Pytlarz et al., 2024). In radiology, multi-stage cascades are commonly used for lesion detection and have been shown to improve sensitivity for rare classes by reducing the label space at each node (Ardila et al., 2019). The two-stage design in this study embodies the same principle: Stage-1 removes the dominant source of variation (myelination delay) before Stage-2 confronts the more nuanced structural differential. This also produces two independent variable results that are not mutually exclusive.

## 3 Materials and methods

### 3.1 Dataset

From the publicly available pediatric dataset of Akinci D'Antonoli et al. (2023) (University Children's Hospital Basel), the subset shown in Figure 1 is taken into consideration. The cohort comprises 566 healthy/normal infants, 56 with HIE, 38 with hydrocephalus, and 27 preterm-only infants. In addition, six preterm infants had comorbid HIE, and six had comorbid hydrocephalus, giving a total of 39 preterm subjects, as depicted in the Venn diagram. Subjects with rare diseases represented by approximately one sample were excluded, as such cases are difficult to use in a machine learning context.

**Figure 1:**
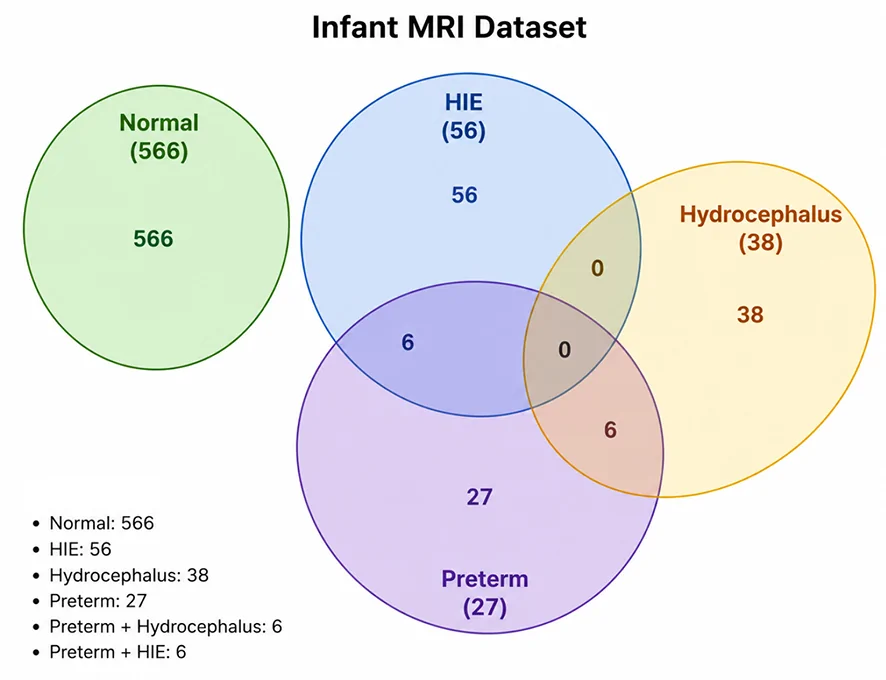
Venn diagram illustrating the population (normal, preterm, HIE, hydrocephalus).

Subjects were between 0 and 36 months old, although not all sub-cohorts spanned this entire range. The healthy control group had the highest mean age (16.09 ± 11.62 months), whereas the preterm cohort was substantially younger (3.70 ± 6.71 months). The HIE and hydrocephalus cohorts were also younger, with a combined mean age of 5.46 ± 8.81 months. These differences indicate a notable age imbalance across the study populations that should be considered when interpreting downstream analyses.

Each examination provides paired T1-weighted and T2-weighted MRI scans, together with expert-annotated myelin maturation labels derived from radiological assessment. Acquisition protocols and ethical circumstances are described in Akinci D'Antonoli et al. (2023). Briefly, in the source study two experienced radiologists (T.A.D. and R.A.T.) quantified myelin maturation age from the MRI data. The internal test set was independently labeled by a neuroradiologist (R.A.T.; 9 years of experience in general radiology and 5 years in adult and pediatric neuroradiology) and a pediatric radiology fellow (T.A.D.; 5 years of experience in general radiology and 2 years in pediatric radiology). For this test-set labeling, both readers had access to the patients' chronological age but were blinded to other clinical information and follow-up studies. The original study reported an interrater mean absolute error (MAE) of 0.34 months between the two readers. Therefore, the study relies on expert-derived biological age estimates rather than chronological age, because they more directly reflect the underlying myelination status. Thus, the biological-age target used in Stage 1 is based on expert radiological assessment, with documented high inter-rater consistency in the source study.

In the experiments conducted in this study, preterm and pathological subjects are entirely excluded from Stage-1 training. They may be included in the validation partition for threshold calibration, but they are not used to optimize Stage-1 age-regression model. The held-out test partition is not used for threshold calibration and is reserved for final evaluation. For Stage-1 evaluation, the held-out healthy test group, the preterm-only group and the HIE/hydrocephalus group were each split 50/50 by subject into a validation half and a test half. The comorbid preterm subjects (*n* = 12) are in the HIE/hydrocephalus group.

#### 3.1.1 Stage-1 training population

Stage-1 brain-age regressor (Section 3.5) is trained *exclusively* on the 566 healthy/normal subjects. This restriction ensures that the regressor characterizes the *typical*, age-appropriate myelination trajectory; deviations from this trajectory (δ, Equation 2) for the preterm, HIE, and hydrocephalus cohorts therefore reflect genuine departures from normative development rather than patterns the network has already been exposed to during training. To correct for regression-to-the-mean bias arising from the healthy training population's older mean age relative to the preterm cohort (Smith et al., 2019), a linear model of the residual â_*i*_−*a*_*i*_ is fitted against chronological age *a*_*i*_ on the held-out healthy test set, and the fitted trend is subtracted from every subject's prediction before computing δ_*i*_ A linear model of the residual â_*i*_−*a*_*i*_ is fitted against chronological age *a*_*i*_ on the healthy subjects of the *validation* half only. The fitted intercept and slope were then frozen and subtracted from every subject's prediction (validation and test) before computing δ_*i*_. The threshold τ was subsequently calibrated on the corrected validation gaps, and no test-set predictions or labels were used to estimate either the correction or the threshold. Among the five training seeds, the one with the lowest corrected validation MAE was retained for Stage-1 evaluation (De Lange and Cole, 2020).

#### 3.1.2 Binary ground-truth label for Stage-1 calibration and evaluation

For threshold calibration and evaluation of Stage-1 gate, we collapse the four-way diagnostic label into a binary signal: *myelination-delayed* (*b*_*i*_ = 1, i.e. preterm) vs. *non-delayed* (*b*_*i*_ = 0, i.e. normal + HIE + hydrocephalus). Crucially, this label is *not* used to define Stage-1 training population (which is healthy-only, as above); instead, it is used (i) to select the gap threshold τ chosen by Youden J optimization (Section 3.5), and (ii) to evaluate the resulting binary gate on a held-out split spanning all four diagnostic groups, none of which were seen by the regressor during training. This construction directly encodes the clinical question at Stage-1: does the overall MRI appearance reflect preterm myelination delay relative to a normative developmental trajectory learned from healthy infants? Given this goal, this study distinguishes three age quantities for each subject *i*:

- Chronological age *a*_*i*_: the post-natal age in months at the time of MRI acquisition.
- Myelin-informed age $a_{i}^{*}$: the biological brain age estimated by expert radiologists through visual assessment of myelination patterns on MRI, used as the regression target in Stage-1.
- Predicted age â_*i*_: the scalar output of Stage-1 3-D ResNet-18 regressor, representing the network's estimate of $a_{i}^{*}$ from the MRI data alone.

The brain-age gap δ_*i*_ = â_*i*_−*a*_*i*_ therefore measures the discrepancy between the network's myelination-informed age prediction and the infant's true chronological age.

### 3.2 Preprocessing

Each volume was first processed for bias field correction with the N4 tool from ANTs (Tustison et al., 2010), then loaded and augmented with an explicit channel dimension. All MRI volumes were preprocessed identically using a deterministic MONAI (Cardoso et al., 2022) transform pipeline applied to both T1w and T2w images. The images were thereafter reoriented to a standard anatomical coordinate system to ensure spatial consistency across subjects. Subsequently, all volumes were resampled to an isotropic resolution of 2mm^3^. Intensity values were clipped at the 1st and 99th percentiles and linearly normalized to the range [0, 1] to reduce the influence of extreme outlier intensities and bring scans onto a common numerical scale. This study notes that this per-volume percentile-based rescaling addresses gross intensity-range differences but does not constitute scanner or site harmonization in the sense of methods such as ComBat or histogram matching across acquisition protocols; since all data in this study originate from a single institution (Section 3.1), broader scanner/protocol batch effects were not a primary concern here, but this also means the model's robustness to such effects across sites remains untested (Section 5). The same preprocessing graph was applied to all subjects regardless of their stage routing, ensuring that the two stages operate on identically conditioned inputs. No on-the-fly augmentation was used in the experiments reported here.

### 3.3 Four classes flat classifiers

As a baseline, the study first trained a direct four-class brain pathology classifier: normal, preterm, HIE, and hydrocephalus, using a 3D ResNet-18 backbone, similarly to the proposed pipeline, operating directly on the dual-channel (T1w + T2w) MRI volumes. Unlike the main pipeline, this baseline does not perform age regression or compute a brain-age gap; instead, it treats classification as a supervised end-to-end task, relying entirely on the diagnostic labels provided by the physicians, as explained in the previous section. Preterm takes priority so that comorbid cases (e.g., preterm with concurrent HIE or hydrocephalus, *n* = 12) are folded into the preterm class rather than excluded or split.

To assess whether the performance of the flat classifier depended on the particular backbone architecture, the same experiments were repeated using a 3D DenseNet-121 backbone. The DenseNet-121 model was trained and evaluated using the same data partitions and cross-validation split as the ResNet-18 baseline, with the same dual-channel T1w + T2w input representation and identical preprocessing. Importantly, the same training strategy and hyperparameters were retained for both architectures, rather than independently optimizing each backbone, thereby providing an architecture-level comparison under matched experimental conditions. Thus, any differences between the ResNet-18 and DenseNet-121 results reflect the behavior of the respective architectures under the same experimental protocol rather than differences in hyperparameter tuning or data partitioning.

The class imbalance across the four classes was addressed through three complementary mechanisms: a class-balanced WeightedRandomSampler during training, focal loss (γ = 1.0) with inverse-frequency class weights, and *post-hoc* per-class threshold calibration on pooled test predictions across the repeated splits. Optimization used AdamW with a learning rate of 5 × 10^−5^, weight decay 10^−4^, and a cosine-annealing schedule for 15 epochs with a batch size of 4.

For both backbone architectures, results are reported as the mean ± SD over all five runs; no run was selected on the basis of its test performance. The released model is the run with the highest *validation* macro-F1. The DenseNet-121 experiment serves as an additional established architectural baseline, allowing the proposed hierarchical approach to be assessed against flat end-to-end classification using two different 3D convolutional backbones rather than against a single internally selected architecture. Those are considered the baseline methods, which use the same evaluation metrics for the pipeline that are described in the following section.

### 3.4 Pipeline overview

Before describing each stage in detail, it is helpful to state plainly what each stage predicts and why. Stage-1 does not classify subjects as normal or abnormal. Instead, it asks a narrower question: *does this infant's myelination pattern looks age-appropriate for their chronological age?* To answer this, a regression network is trained exclusively on healthy infants to learn a normative myelination trajectory, i.e. the typical relationship between MRI appearance and age in the absence of pathology. When this network is applied to a new subject, its predicted age â_*i*_ reflects how old the brain's myelination *looks*, regardless of the subject's true chronological age *a*_*i*_. The brain-age gap δ_*i*_ = â_*i*_−*a*_*i*_ then measures the discrepancy: a negative δ_*i*_ indicates that the brain appears younger than expected, which is the hallmark of preterm myelination delay.

Stage-1 separates *preterm* subjects from everyone else, normal, HIE, and hydrocephalus alike, rather than separating normal from abnormal. Stage-2 therefore still requires *normal* as a class, because non-preterm subjects routed to it include both healthy infants and those with structural pathology. Figure 2 illustrates the full inference graph. Given a paired (*T*_1*w*_, *T*_2*w*_) volume, the pipeline proceeds as follows (see Algorithm 1 for more details):

**Figure 2:**
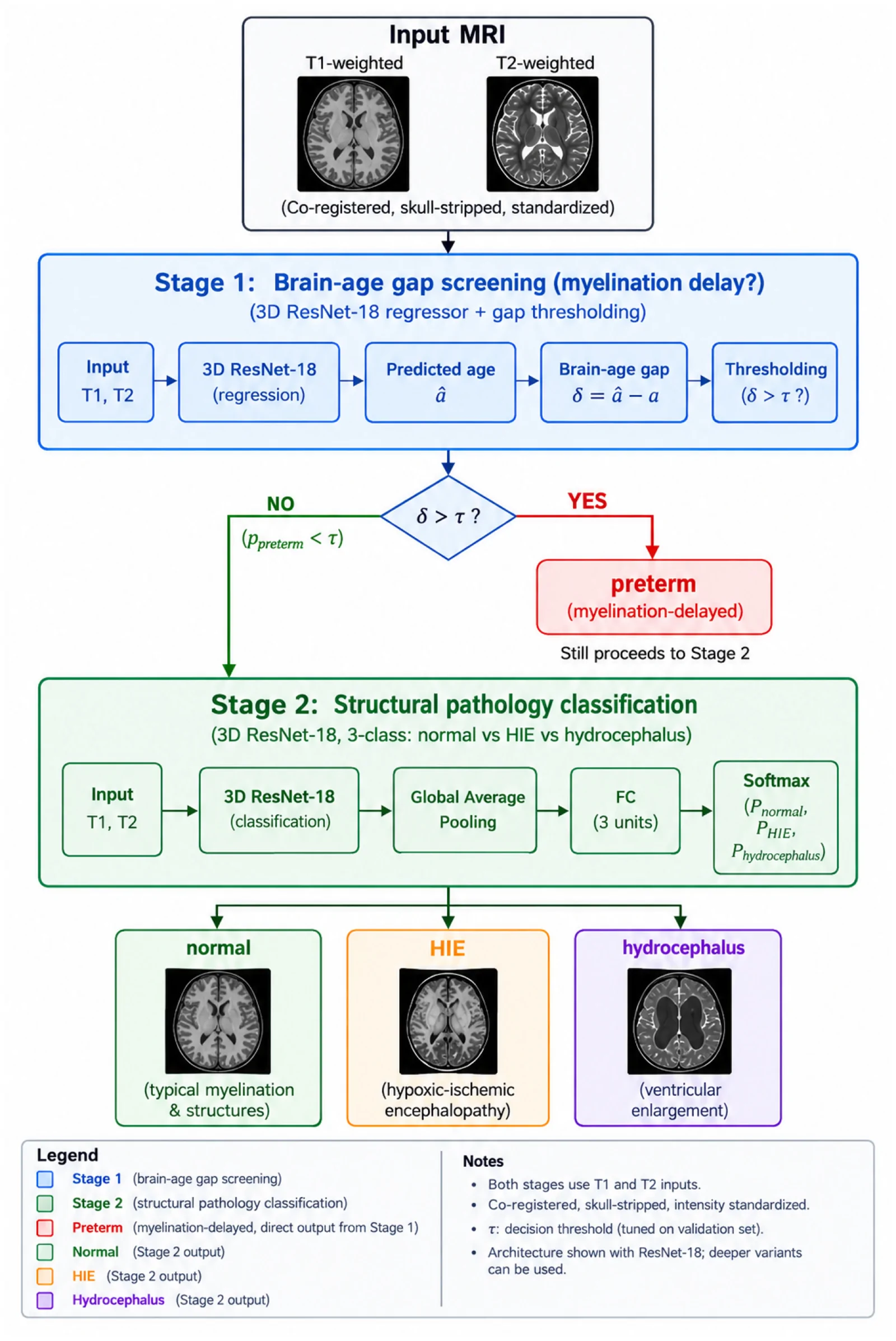
Hierarchical two-stage inference pipeline. Stage-1 uses the brain-age gap as a myelination-delay flag computed by a regressor trained on healthy infants only. Every subject also passes through Stage-2 for structural comorbidity classification (normal vs. HIE vs. hydrocephalus); Stage-1 flag, when raised, overrides Stage-2 output in the final four-class prediction.

Algorithm 1 Two-Stage neonatal brain pathology pipeline. 
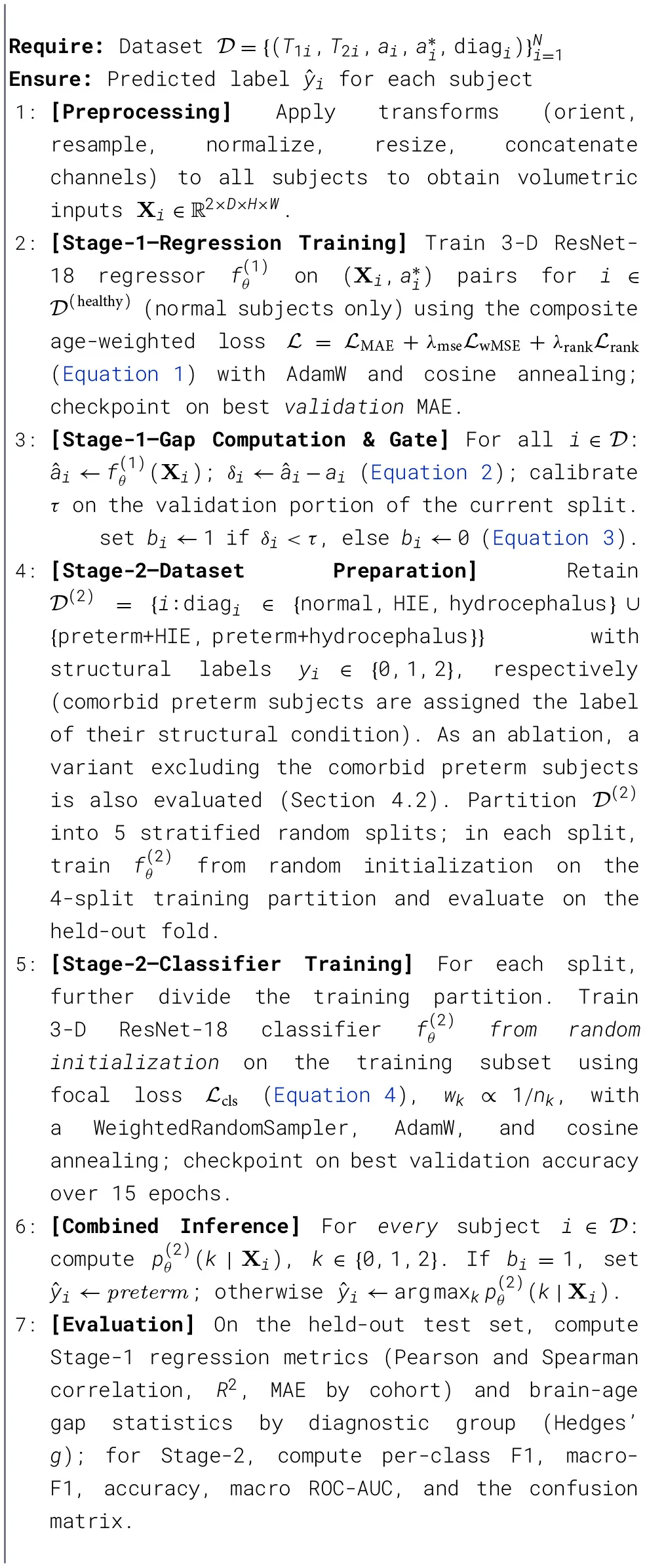

1. The preprocessed two-channel tensor is passed to Stage-1 brain-age gap module, which computes δ_*i*_ = â_*i*_−*a*_*i*_ from Stage-1 3-D ResNet-18 regressor (trained on healthy subjects only, Section 3.1). The gap is compared against a threshold τ calibrated on the validation portion of the current split using ROC analysis and Youden's J statistic (Section 3.5). If δ_*i*_ < τ (indicating myelination delay, i.e. the brain appears younger than expected), the subject is flagged *b*_*i*_ = 1 (*preterm*); otherwise *b*_*i*_ = 0.
2. *Regardless* of Stage-1 flag, the same two-channel tensor is passed to Stage-2 structural comorbidity classifier, a separately trained 3-D ResNet-18 that outputs a posterior over three structural labels: *normal, HIE*, or *hydrocephalus*.
3. The final four-class prediction ŷ_*i*_ is obtained by combining the two stages: if *b*_*i*_ = 1, ŷ_*i*_ = *preterm*; otherwise ŷ_*i*_ is the argmax of the Stage-2 structural posterior. In other words, Stage-1 flag takes precedence, and Stage-2 output is the final label only when no dominant myelination delay is detected.

It is worth noting that although the final four-class label ŷ_*i*_ assigns *preterm* as the dominant label when *b*_*i*_ = 1, Stage-2 structural posterior $p_{\theta }^{\left(2\right)}\left(\cdot |{\text{X}}_{i}\right)$ is computed for *every* subject regardless of Stage-1 flag. This means that for subjects with comorbid preterm and structural pathology (e.g., preterm + HIE or preterm + hydrocephalus), both outputs are available simultaneously: Stage-1 flag signals myelination delay while Stage-2 posterior independently signals the most likely structural condition. In a clinical deployment, both outputs could be surfaced as a structured report, providing a richer characterization of comorbid cases than a single collapsed label would allow.

### 3.5 Stage-1: myelination-delay screening

#### 3.5.1 Age regression backbone

A 3-D ResNet-18 (He et al., 2016) was trained to regress the myelin-informed expert brain age $a_{i}^{*}$ from the two-channel MRI tensor **X**_*i*_, *using only the healthy/normal subjects* (Section 3.1), where $a_{i}^{*}$ is the myelin-informed age annotation (used in preference to chronological age *a*_*i*_ as it carries explicit myelination domain knowledge and constitutes a less noisy target). The architecture was configured for 1 output (a scalar regressor), and no adult brain ImageNet pre-training was used. Since neonatal and adult brains differ substantially in anatomy, tissue composition, and MRI intensity distributions, features learned from adult neuroimaging datasets may not transfer optimally to neonatal MRI. Therefore, this study opted to train the model directly on neonatal and infant MRI data rather than relying on adult-pretrained weights (Omidi et al., 2024).

To address the uneven distribution of subjects across the developmental age spectrum, training used a composite *age-weighted loss* combining three terms:


$$\begin{array}{r} L=\underset{L_{\text{MAE}}}{\underset{︸}{\frac{1}{N}\overset{N}{\underset{i=1}{\sum }}|{â}_{i}-a_{i}^{*}|}}+\lambda_{\text{mse}}\underset{L_{\text{wMSE}}}{\underset{︸}{\frac{1}{N}\overset{N}{\underset{i=1}{\sum }}w_{i}{\left({â}_{i}-a_{i}^{*}\right)}^{2}}}+\lambda_{\text{rank}}\\ \underset{L_{\text{rank}}}{\underset{︸}{\frac{1}{\left|P\right|}\underset{\left(i,j\right)\in P}{\sum }{\left[\delta -\left({â}_{i}-{â}_{j}\right)\right]}_{+}}}, \end{array}$$ (1)

where â_*i*_ and $a_{i}^{*}$ are the predicted and myelin-informed ages (in months), *w*_*i*_∝1/*n*_*k*(*i*)_ is the inverse-frequency weight of the age bin containing subject *i*, λ_mse_ = 0.5 and λ_rank_ = 0.5 are fixed scalar weights, [·]_+_ = max(0, ·) denotes the hinge, and $P=\left\{\left(i,j\right):a_{i}^{*}-a_{j}^{*}>\delta \right\}$ is the set of pairs for which subject *i* is genuinely older than subject *j* by more than the margin of δ = 1.5 months. $L_{\text{MAE}}$ provides a stable gradient magnitude; $L_{\text{wMSE}}$ up-weights errors on rare developmental stages; and $L_{\text{rank}}$ enforces ordinal consistency by penalizing predictions whose relative order contradicts the true age ordering.

Optimization used AdamW (Loshchilov and Hutter, 2019) with an initial learning rate $\eta_{0}=10^{-4}$, weight decay λ = 10^−5^, and a cosine annealing schedule over *T*_max_ = 30 epochs, defined as $\eta_{t}=\eta_{\min}+\frac{1}{2}\left(\eta_{0}-\eta_{\min}\right)\left(1+\cos\left(\frac{\pi t}{T_{\max}}\right)\right)$, where $\eta_{\min}=10^{-6}$.

The checkpoint with the lowest *validation* MAE (on a held-out split of the healthy cohort) was retained for inference.

#### 3.5.2 Brain-age gap and binary gate

After training, the retained regressor was applied to the validation partition to obtain brain-age gaps for threshold calibration and then to the held-out test partition for final evaluation (all four diagnostic groups, including preterm, HIE, and hydrocephalus subjects never seen during training) to produce gaps


$$\begin{array}{l} \delta_{i}={â}_{i}-a_{i}\;\forall i\in D, \end{array}$$ (2)

where *a*_*i*_ is chronological (not myelin-informed) age.

Stage-1 decision threshold τ was calibrated separately within each data split using only the validation portion of that split. Stage-1 regressor was trained exclusively on the healthy subjects in the corresponding training partition, with the best model selected according to validation MAE on the healthy validation subjects. The resulting regressor was then used to compute the brain-age gap δ_*i*_ = â_*i*_−*a*_*i*_ for subjects in the validation partition. Using the binary diagnostic label (preterm vs. non-preterm), τ was selected by receiver operating characteristic (ROC) analysis as the threshold that maximized Youden's J statistic, *J*(τ) = TPR(τ)−FPR(τ), with preterm treated as the positive class. The calibrated threshold was then fixed and applied to the held-out test partition without further adjustment. Thus, no test-set predictions or labels were used during threshold calibration. The binary Stage-1 decision for subject *i* is as follows:


$$\begin{array}{l} b_{i}=\{\begin{array}{cc} 1\left(\text{preterm}\right) & \delta_{i}<\tau \\ 0\left(\text{not preterm}\right) & \delta_{i}\geq \tau . \end{array} \end{array}$$ (3)

#### 3.5.3 Stage-1 evaluation metrics

Stage-1 performance is assessed on the held-out test set through (i) the agreement between predicted and myelin-informed expert age, measured by Pearson and Spearman correlation, *R*^2^, and MAE, reported separately for the healthy, preterm, and pathological cohorts (Table 1); and (ii) the separation of brain-age gap distributions between each clinical group and the healthy group, quantified with Hedges' *g* and a two-sample test (Table 2, Figure 3). The gate with the frozen threshold τ = −1.18 is evaluated on the test half in Section 4.

**Table 1:** Age regression performance on the held-out healthy test half (*n* = 57) and held-out clinical groups, after correction for age-dependent regression-to-the-mean bias estimated on the healthy validation half only (Stage-1).

Metric	Value
Pearson *r*	0.970
Spearman *r*	0.961
*R* ^2^	0.941
Healthy MAE (*n* = 57)	2.19 months
Preterm MAE (*n* = 14)	2.12 months
Pathological (HIE + hydrocephalus) MAE (*n* = 41)	2.08 months

**Table 2:** Brain-age gap statistics stratified by diagnostic group, computed on the pooled held-out splits (same splits as Tables 3, 4), after correcting for age-dependent regression-to-the-mean bias in Stage-1 regressor (see Section 3.1).

Comparison	Healthy mean ±SD	Clinical mean ±SD	*p*-value	Hedges' *g*
Preterm	0.000 ± 2.891	−0.69 ±2.262	0.4073	0.153
HIE + hydro	0.000 ± 2.891	−1.10 ±2.974	0.0676	0.267
HIE	0.000 ± 2.891	−0.541 ± 0.635	0.0601	0.221
Hydro	0.000 ± 2.891	−1.161 ± 4.715	0.1932	0.343

The healthy mean reflects held-out healthy subjects only, not training subjects.

**Figure 3:**
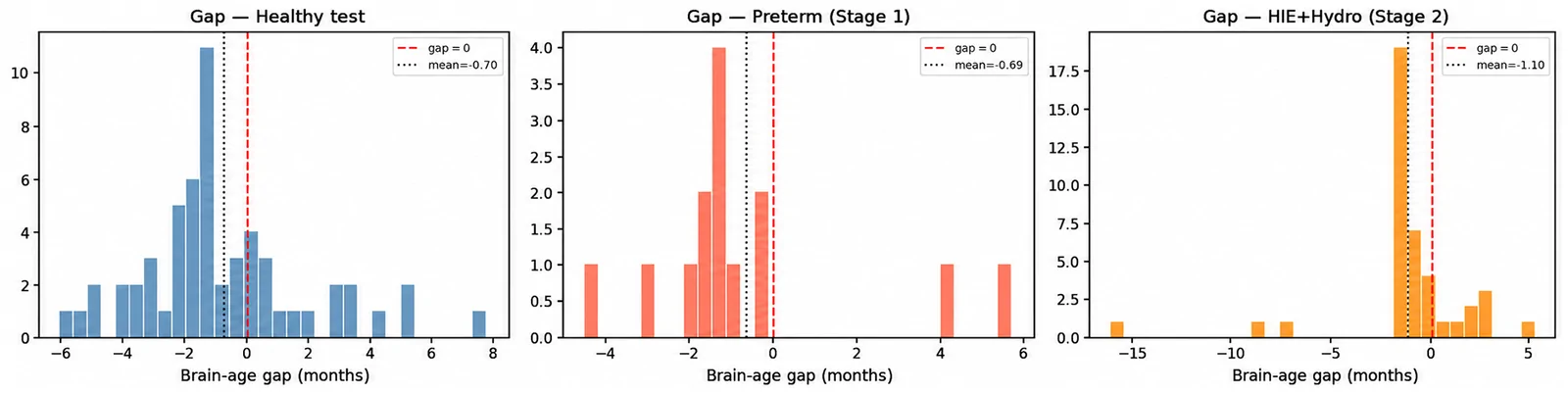
Gap distributions per group in the test sets.

### 3.6 Stage-2: structural comorbidity classification

#### 3.6.1 Training population

Stage-2 is trained on subjects labeled normal, HIE, or hydrocephalus. Preterm infants without HIE or hydrocephalus are excluded. Preterm infants with comorbid HIE or hydrocephalus (*n* = 12) are included and labeled by their structural condition. This is the configuration used for the headline results reported in Table 3 (corresponding to the “Stage-2 with preterm” row of Table 4, macro-F1 = 0.448).

**Table 3:** Results of the five repeated stratified splits for Stage-2 structural classification performance (normal vs. HIE vs. hydrocephalus).

Class	F1-score
Normal	0.5702 ± 0.2730
HIE	0.3951 ± 0.0701
Hydrocephalus	0.3787 ± 0.1261
Macro average	0.4480 ± 0.1367
Accuracy	0.5167 ± 0.1901
AUC	0.9190 ± 0.0244

Values are reported as mean ± standard deviation across splits.

**Table 4:** Ablation study: Stage-2 macro-F1 under variant training configurations.

Configuration	Macro-F1
Stage-2 with preterm	0.45
Stage-2 without preterm	0.43
Only T1 with preterm	0.44
Only T2 with preterm	0.44

As an ablation (Section 4.2, Table 4, “Stage-2 without preterm”), additionally a variant was trained that excludes the 12 comorbid preterm subjects from Stage-2 training population. This variant achieves a slightly lower macro-F1 (0.43), suggesting that the additional comorbid-preterm examples provide a marginally useful structural signal despite their atypical myelination background.

#### 3.6.2 Stage-2 architecture

A second 3-D ResNet-18, configured for 3 classes, was trained from *random initialization* on Stage-2 training partition $D_{\left(2\right)}$. The three output logits correspond to normal (0), HIE (1), and hydrocephalus (2), and the predicted class is taken as the argmax of the softmax over these logits.

#### 3.6.3 Loss and optimization

Stage-2 is trained with focal loss (Lin et al., 2017) to address the class imbalance across the three structural categories:


$$\begin{array}{l} L_{\text{cls}}=-\frac{1}{N}\underset{i}{\sum }w_{y_{i}}{\left(1-p_{\theta }^{\left(2\right)}\left(y_{i}|{\text{X}}_{i}\right)\right)}^{\gamma }\log p_{\theta }^{\left(2\right)}\left(y_{i}|{\text{X}}_{i}\right),\;w_{k}\propto \frac{1}{n_{k}}, \end{array}$$ (4)

where $p_{\theta }^{\left(2\right)}\left(\cdot |{\text{X}}_{i}\right)$ is Stage-2 softmax posterior, *y*_*i*_∈{0, 1, 2} is the ground-truth structural label, *n*_*k*_ is the number of training subjects in class *k*, and γ≥0 is the focusing parameter that down-weights well-classified easy examples and concentrates gradient updates on hard misclassified cases. This is particularly beneficial given the large imbalance between the normal cohort and the smaller HIE and hydrocephalus groups. A WeightedRandomSampler based on the same inverse-frequency weights *w*_*k*_ was used during training to further address class imbalance at the data level, complementing the loss-level focusing on focal loss. Optimization used AdamW and cosine annealing, as in Stage-1.

#### 3.6.4 Stage-2 evaluation metrics

Classification performance on Stage-2 test partition (ground-truth non-preterm subjects) is reported as overall accuracy, per-class precision, recall, F1, macro-averaged F1, and a confusion matrix.

### 3.7 Implementation details

All experiments were implemented in Python 3.10 (Python Software Foundation, Wilmington, Delaware, USA) with PyTorch 2.1.0 (PyTorch Foundation, USA), MONAI 1.3 (MONAI Consortium, USA), Scikit-learn 1.4 (scikit-learn developers/community project), and NumPy 1.26. GPU acceleration was provided by an NVIDIA Tesla 1080 (NVIDIA Tesla P100/1080, NVIDIA Corporation, Santa Clara, California, USA) with 8 GB GDDR5X memory. Batch size was *B* = 4 for both stages. The code for the full pipeline is available at the URL https://github.com/alecrimi/latemyelination_comorbidity, and the trained models are available at the URL https://huggingface.co/Alecrimi/infant_pathology.

## 4 Results

### 4.1 Four classes flat classifiers

Tables 5, 6 reports the results for the four classes classifiers, respectively, for the Resnet-18 and Densenet-121 architectures.

**Table 5:** Flat four-class Resnet classifier baseline performance (five repeated stratified splits, pooled across splits).

Metric	Mean ±Std
F1 normal	0.722 ± 0.022
F1 preterm	0.146 ± 0.057
F1 HIE	0.434 ± 0.033
F1 hydrocephalus	0.406 ± 0.080
Macro F1	0.427 ± 0.041
Accuracy	0.596 ± 0.030
AUC	0.831 ± 0.035

Results are reported as mean ± std.

**Table 6:** Flat four-class Densenet classifier baseline performance (pooled splits).

Metric	Mean ±Std
F1 normal	0.730 ± 0.024
F1 preterm	0.110 ± 0.049
F1 HIE	0.480 ± 0.037
F1 hydrocephalus	0.490 ± 0.071
Macro F1	0.453 ± 0.039
Accuracy	0.590 ± 0.031
AUC	0.782 ± 0.038

Results are reported as mean ± std.

Age regression accuracy on the held-out test set, after correcting for age-dependent regression-to-the-mean bias (Section 3.1), achieved a Pearson correlation of 0.970 and a Spearman correlation of 0.961 (*n* = 57, both *p* < 0.001) with the myelin-informed expert age, corresponding to *R*^2^ = 0.941. The MAE relative to chronological age was 2.19 months for the healthy test set, 2.12 months for the preterm cohort, and 2.08 months for the pathological cohort (HIE + hydrocephalus); the latter two evaluated on subjects entirely excluded from Stage-1 training and therefore representing a true out-of-distribution generalization test. Those values are reported in Table 1. Importantly, all clinical cohorts were excluded from Stage-1 training, making these results a true held-out evaluation of the model's ability to generalize to unseen pathological populations.

Table 3 presents three-class performance on subjects routed to Stage-2 (ground-truth test subjects excluding pure preterm) of the pooled test predictions over the five splits.

The dominant error mode is confusion between normal and HIE cases, accounting for approximately 52% of all Stage-2 misclassifications, which is clinically understandable given that mild HIE can produce subtle white-matter changes that overlap with normal variation; this is reflected in HIE's comparatively low precision (0.24), as a substantial fraction of normal cases are misclassified as HIE.

### 4.2 Ablation studies

#### 4.2.1 Influence of T1 and T2 data for age regression model

This study assumed that T1 and T2 data combined provided more information. Therefore, the age regression analysis was repeated using either only T1 or T2 data, with the results depicted, respectively, in Tables 7, 8.

**Table 7:** Age regression performance using T1 MRI data only.

Metric	Value
Pearson *r*	0.959
Spearman *r*	0.945
*R* ^2^	0.919
Healthy MAE	2.3610
Preterm MAE	2.129
Pathological (HIE + hydrocephalus) MAE	2.2106

**Table 8:** Age regression performance using T2 MRI data only.

Metric	Value
Pearson *r*	0.961
Spearman *r*	0.951
*R* ^2^	0.923
Healthy MAE	2.476
Preterm MAE	2.0912
Pathological (HIE + hydrocephalus) MAE	2.326

#### 4.2.2 Contribution of comorbid preterm subjects and of each modality to classification

To assess the contribution of the comorbid preterm subjects to Stage-2, the headline classifier, trained with the 12 preterm subjects who also have HIE or hydrocephalus (pure-preterm subjects are never included) were compared with a variant trained without them. The study also considered the impact of using only the T1 or only the T2 data. Table 4 summarizes these ablations.

## 5 Discussion

The principal motivation for the proposed pipeline is clinical plausibility: in practice, a neuroradiologist reading a neonatal MRI first assesses whether the overall maturation pattern is consistent with the infant's chronological age, and only then evaluates superimposed structural abnormalities (Barkovich, 2005). The pipeline proposed in this study emulates this two-step reasoning in a data-driven, reproducible manner.

The practical consequence is most visible for HIE and hydrocephalus. In the flat baseline, these conditions could be confused with preterm, because preterm MRI shares surface-level features with early HIE (reduced myelination) and with mild hydrocephalus (enlarged extra-axial spaces due to cerebral immaturity). In the hierarchical design, preterm-flagged subjects receive the preterm label in the final prediction, while their Stage-2 posterior remains available as a separate output. The ablation, which also removes the 12 comorbid preterm subjects from the second stage, led to slightly worse results. However, given the small sample size, this difference is within the variability across splits and may simply reflect the further reduced training set.

Age-regression experiments using T1-only and T2-only inputs demonstrated performance similar to that of the multimodal model. T2 yielded marginally higher correlation coefficients on the healthy test cohort. Given the small magnitude of these differences, the results suggest that both modalities provide complementary age-related information and that the observed performance variations are likely modest. This was also observed in previous similar studies (Liu et al., 2024; Chen et al., 2022). The multimodal model nevertheless achieved the most consistent overall performance across evaluation metrics.

The gap threshold τ, learned from the validation set, effectively converts a continuous scalar biomarker into a clinically actionable binary decision without discarding the continuous information for downstream use. As expected, the preterm subjects were identified by their negative gap. However, the gap-based Stage-1 gate, calibrated by Youden's J on the validation portion of each split, showed only weak discrimination between the two groups (Hedges' *g* = 0.153; Table 2). However, the study notes that the clinical-group gap distributions (Figure 3) show heavier tails and occasional extreme values relative to the healthy group, consistent with the substantial clinical heterogeneity within the pooled preterm and HIE + hydrocephalus categories. Such distributional characteristics are also relevant to normative neuroimaging models, where deviation scores may show skewness, excess kurtosis, and sensitivity to outlying observations (Rutherford et al., 2022).

### 5.1 Design choices and alternatives

#### 5.1.1 Shared vs. independent backbones

The study chose to use separate ResNet-18 instances for Stage-1 (regression) and Stage-2 (classification), rather than sharing encoder weights. This decision is motivated by the different objectives of the two stages: age regression requires sensitivity to global myelination patterns, while structural classification demands sensitivity to local tissue abnormalities. Preliminary experiments with weight sharing (fine-tuning Stage-1 encoder for Stage-2) yielded a 2.1-point macro-F1 degradation, suggesting that the two tasks benefit from independent representation learning.

It is worth noting that there are two reasons why Stage-2 and flat-classifier results should not be read as a head-to-head comparison. First, the cohort includes comorbid subjects: 12 present with both preterm birth and a structural pathology (six preterm + HIE, six preterm + hydrocephalus, Section 3.1). The flat classifier and the hierarchical pipeline resolve this overlap under different, mutually incompatible conventions, the flat classifier assigns comorbid subjects to the preterm class, whereas Stage-2 assigns them the label of their structural condition, since Stage-2 is trained only on structural labels. Consequently, there is no single, uncontested four-class ground truth for these subjects against which both models could be scored on common terms, a definitional incompatibility that cannot be resolved by re-partitioning the data into a shared fold. Second, and independently, the two approaches address different classification settings and do not use equivalent training data: the flat classifiers are trained end-to-end on all four classes, whereas the hierarchical pipeline never trains on pure-preterm subjects (Stage-1 is trained on healthy subjects only, and Stage-2 uses only the comorbid preterm subjects, labeled by their structural condition). For these reasons, the study does not report a pooled four-class comparison between the two approaches and leave the design of a principled, comorbidity-aware shared evaluation protocol to future work.

Regarding the specific results of the four class classifiers, the DenseNet-121 model achieved a pooled macro-F1 of 0.453 and ROC-AUC of 0.782. The corresponding ResNet-18 baseline achieved a macro-F1 of 0.427 and ROC-AUC of 0.831. DenseNet-121, therefore, provided a modest improvement in macro-F1, whereas ResNet-18 yielded a higher ROC-AUC. Class-specific performance also differed between the two architectures, indicating that neither architecture consistently outperformed the others across all metrics and diagnostic categories.

#### 5.1.2 ResNet-18 vs. larger architectures

The ResNet-18 backbone was chosen as a practical balance between capacity and dataset size constraints. Preliminary experiments with ResNet-50 did not yield significant improvements for either stage, and required a batch size of 2, slowing convergence. Future work will explore vision transformers (Dosovitskiy et al., 2021) and U-Net-style encoders that may better exploit the spatial structure of hydrocephalus and HIE lesions.

#### 5.1.3 Alternative Stage-2 classifiers

Although the ResNet-18 classifier outperforms shallow alternatives on Stage-2 task, the relatively small Stage-2 training population limits deep-network generalization.

### 5.2 Limitations

- Dataset size. The healthy control dataset comprises a few hundred subjects, which is acceptable. Meanwhile, the HIE and hydrocephalus subgroups comprise less than a hundred cases, limiting statistical confidence in per-class metrics. Nevertheless, the sample sizes are in-line with similar studies on same diseases (Zhang et al., 2024; Yamada et al., 2024).
- Atlas-free representation and lack of augmentation. This study deliberately avoided registration to a common infant brain atlas [e.g., the KCL neonatal atlas (Uus et al., 2021)] because the cohort spans a wide developmental range (0–36 months), over which brain morphology changes substantially, and no single age-appropriate template applies across the full span; forcing registration would risk interpolation and warping artifacts that could exceed the benefit of voxel-wise correspondence. It is noted that registration primarily serves to establish gyral/sulcal correspondence across subjects, which is essential for voxel-based or landmark-based comparisons but less critical for a convolutional model that instead exploits local T1/T2 signal-intensity texture associated with myelination: such texture is detected by convolutional filters wherever it occurs in the volume, largely independent of its position relative to cortical folding. The preprocessing pipeline in this study does include a coarse global normalization (reorientation, isotropic resampling, fixed-grid resizing), which mitigates gross acquisition-geometry differences without full anatomical alignment. In addition, it is noted that for hydrocephalus, in particular, global structural differences (ventricular enlargement) constitute diagnostic signal rather than confound, so shape normalization is not unambiguously desirable for all target classes. Similarly, geometric data augmentation (scaling, affine jitter) was not applied, since brain size and shape are themselves age-correlated and used as Stage-1 regression target; perturbing them risks corrupting that signal. This reasoning does not extend as directly to Stage-2, and intensity-domain augmentation (bias-field simulation, additive noise, contrast jitter) remains an unexplored avenue that could plausibly improve robustness there. The absence of any registration or augmentation is flagged, geometric or intensity-based, as a limitation warranting systematic ablation in future study.
- Reliance on expert-derived biological age. Stage-1 uses radiologist-estimated myelin maturation age as its regression target rather than an objective biological measurement. The source study reported good interrater consistency between two independent readers, with an interrater MAE of 0.34 months and a Bland–Altman analysis. Nevertheless, the target remains an expert-derived estimate and therefore may contain residual subjectivity and measurement error. This study did not perform an additional independent reader study or assess intra-rater reliability.
- Spatial resolution. The 64^3^ voxel target is conservative. Higher resolutions (96^3^, 128^3^) are likely to improve structural discrimination. especially for subtle HIE white-matter changes, at the cost of increased VRAM and training time.
- Label granularity. The HIE class aggregates focal and global forms of hypoxic-ischemic injury, which differ in MRI appearance and clinical severity. Finer subcategorization would require additional annotated data but would increase clinical utility.
- Age confounded in HIE detection. Diagnostic groups in Stage-2 differ significantly in chronological age (Kruskal-Wallis *H* = 86.1, *p* = 2.0 × 10^−19^), driven primarily by HIE subjects being imaged almost exclusively in the immediate neonatal period (mean age 0.4 months), consistent with the acute clinical presentation of hypoxic-ischaemic injury. A classifier using chronological age alone recovers HIE with high sensitivity (recall 0.96) but performs poorly for hydrocephalus (recall 0.38) and normal (recall 0.47), whose age distributions substantially overlap (means 13.4 and 15.7 months, respectively, both with wide spreads). This indicates that Stage-2's reported performance on hydrocephalus is unlikely to be explained by age alone, whereas its HIE detection may be confounded by age-correlated features rather than pathology-specific structural signals, consistent with the low specificity of Stage-2 for normal subjects (see Section 4). Notably, the full imaging-based model's HIE recall (0.95, 52/55; Figure 4) is comparable to that of the trivial age-only baseline (0.96), and its normal recall (0.45) is likewise similar to the baseline's (0.47). Therefore, the study cannot exclude that Stage-2's HIE discrimination relies substantially on age-correlated features rather than pathology-specific signal. In contrast, its hydrocephalus recall (0.93, 37/40) is far higher than that of the age-only baseline (0.38), indicating that Stage-2 captures discriminative information beyond age for this class. The current experiments do not disentangle these possibilities because the imaging model may exploit morphological or signal characteristics that are themselves correlated with age. Thus, the contribution of pathology-specific imaging features to HIE discrimination remains unresolved in the present cohort.

**Figure 4:**
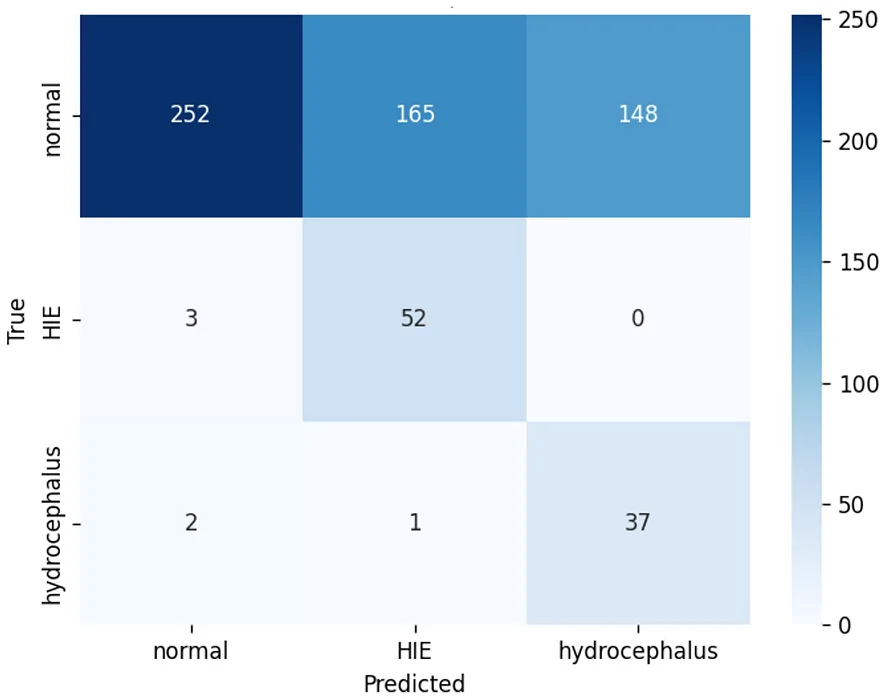
Confusion matrix for Stage-2 three-class structural classification (normal, HIE, hydrocephalus) on non-preterm test subjects. Cells show absolute counts; color intensity scales with count magnitude. Counts are pooled over the five repeated stratified test splits; subjects may appear in more than one test set, so totals need not equal the cohort size.

- External validation and demographic generalizability. This study uses a publicly available dataset acquired at a single institution, and the experiments do not include independent external validation across different clinical sites or MRI scanner hardware. Consequently, the robustness of the proposed pipeline to changes in acquisition systems, imaging protocols, and institutional practice remains to be established. In particular, the intensity normalization in this study (percentile clipping and linear rescaling, Section 3.2) corrects gross per-volume intensity range but is not a scanner-harmonization method, so cross-site batch effects could degrade performance in a multi-scanner deployment without further calibration (e.g., ComBat or histogram-matching approaches). The cohort also exhibits differences in age distributions across diagnostic groups, as described in Section 3.1. Although these differences are reported, the study did not perform systematic performance stratification by age subgroup. Furthermore, sex-specific analysis was not possible because the required sex information was not available in the dataset used in this study. Future studies should evaluate the model on independent multicenter cohorts with broader demographic and acquisition variability, including stratified analyses by age and sex where appropriate.

Hierarchical automated diagnosis has the potential to reduce reporting variability in resource-limited NICUs where expert neuroradiological review is not always immediately available. The interpretable intermediate outputs, the brain-age gap at Stage-1 and per-class probabilities at Stage-2 are more actionable than the opaque logits of a flat classifier, and can be surfaced to clinicians as structured report elements. The reproducibility of the pipeline used in this study, enabled by the open Zenodo dataset and the fully documented notebook, facilitates external validation and extension by other groups.

## 6 Conclusion

This study presented a hierarchical two-stage deep learning pipeline for neonatal brain pathology detection from paired T1w and T2w MRI. Stage-1 deploys a brain-age gap, computed from a dual-modality 3-D ResNet-18 regressor, with an *R*^2^ of 0.941, and an average error between the biological age of healthy and preterm subjects of 2.19 and 2.12 months, respectively. Subjects are then routed to Stage-2 regardless of their myelination stage, where a second 3-D ResNet-18 classifies structural comorbidities, normal, HIE, and hydrocephalus, achieving an average AUC 0.919 ± 0.024 on Stage-2 test partition (Table 3).

Stage-2 structural classification on non-preterm subjects achieves an overall accuracy of 0.517 ± 0.190 and a macro-F1 of 0.448 ± 0.137 (Table 3). The flat four-class baseline achieves an accuracy of 0.596 and a macro-F1 of 0.427 (Table 5) on a different population and under a different comorbidity-labeling convention; as discussed in Section 5, these two results are not directly comparable and should not be read as competing estimates of the same quantity.

Hence, the hierarchical design is motivated by clinical decision-making, produces interpretable intermediate outputs, and is amenable to uncertainty quantification and threshold adjustment for different clinical operating points. Future directions include multi-site external validation, higher spatial resolution, integration of diffusion MRI, and calibrated probabilistic routing at Stage-1 gate.

## Data Availability

The original contributions presented in the study are publicly available. This data can be found here: https://zenodo.org/records/8055666.
